# The Effect of Stacking Sequence on Fatigue Behaviour of Hybrid Pineapple Leaf Fibre/Carbon-Fibre-Reinforced Epoxy Composites

**DOI:** 10.3390/polym13223936

**Published:** 2021-11-15

**Authors:** Mohd Khairul Rabani Hashim, Mohd Shukry Abdul Majid, Mohd Ridzuan Mohd Jamir, Farizul Hafiz Kasim, Mohamed Thariq Hameed Sultan, Ain Umaira Md Shah, Kamarul Arifin Ahmad, Adi Azriff Basri

**Affiliations:** 1 Faculty of Mechanical Engineering Technology, Universiti Malaysia Perlis (UniMAP), Kangar 02100, Malaysia; khairulrabani@unimap.edu.my (M.K.R.H.); ridzuanjamir@unimap.edu.my (M.R.M.J.); 2 Faculty of Chemical Engineering Technology, Universiti Malaysia Perlis (UniMAP), Kangar 02100, Malaysia; farizul@unimap.edu.my; 3 Frontier Materials Research, Centre of Excellence (FrontMate), Universiti Malaysia Perlis (UniMAP), Kangar 02100, Malaysia; 4 Department of Aerospace Engineering, Faculty of Engineering, Universiti Putra Malaysia, Serdang 43400, Malaysia; aekamarul@upm.edu.my (K.A.A.); adiazriff@upm.edu.my (A.A.B.); 5 Laboratory of Biocomposite Technology, Institute of Tropical Forestry and Forest Products (INTROP), Universiti Putra Malaysia, Serdang 43400, Malaysia; 6 Aerospace Malaysia Innovation Centre (944751-A), Prime Minister’s Department, MIGHT Partnership Hub, Jalan Impact, Cyberjaya 63000, Malaysia

**Keywords:** PALF, carbon fibre, hybrid composite, tensile properties, fatigue behaviour

## Abstract

This study examined the fatigue behaviour of pineapple leaf fibre/carbon hybrid laminate composites under various stacking sequences. The vacuum infusion technique was used to fabricate the symmetric quasi-isotropic oriented laminates, in which the stacking was varied. The laminate was tested under static and fatigue tensile load according to ASTM D3039-76 and ASTM D3479-96, respectively. Maximum tensile strength and modulus of 119.34 MPa and 6.86 GPa, respectively, were recorded for the laminate with external PALF ply and internal carbon ply oriented at [± 45°_2_, 0°/90°_2_]_s_ (PCCP_45090). The fatigue tests showed that PCCP_45090 and CPPC_09045 (with internal PALF ply and external carbon ply oriented at [0°/90°_2,_ ± 45°_2_]_s_) exhibited a higher useful life, especially at the high-stress level of the ultimate tensile strength. The normalised stress against the number of cycles showed that the stacking sequences of different ply orientations affected the fatigue behaviour more than the stacking sequences of the material. The laminate stacking sequence significantly affected the hysteresis energy and stiffness evolution. The scanning electron microscopy images showed that the fatigue failure modes included fibre pull-out, fibre breakage, matrix cracking, debonding, and delamination. The study concluded that PCCP_45090 exhibited an outstanding fatigue performance.

## 1. Introduction

Advanced industries, including the mechanical, aerospace, marine, and military industries, are driving fibre-reinforced composite technology. Compared to other fibre-reinforced polymers, carbon-fibre-reinforced polymer (CFRP) materials have been preferentially selected as structural components owing to their superior mechanical properties. Although CFRP has superior physical and mechanical properties, its major drawback is its brittle behaviour and high production costs [1]. CFRP cannot be easily decomposed and recycled, leading to changes to improve its environmental sustainability and recyclability. Through hybridisation with natural fibres, the hybrid composite is more tolerant to brittle failure, minimises the overall production cost, and is environmentally friendly. Natural fibres have unique properties, such as low density, low weight, high specific properties, low cost, and recyclability. Integrating the unique properties of both carbon and natural fibre, the hybrid composite has relatively low mechanical, physical, and thermal properties, as compared to CFRP. The tailored fabrication of the structural design of the composite enhances the mechanical and physical properties of the hybrid composite to satisfy specific applications. Tailoring stacking sequences is a promising method to enhance the aforementioned properties.

Varying the stacking sequence of the fibre-reinforced polymer composite manipulates the mechanical properties and damage behaviour, which imparts the expected anisotropic behaviour. Zhang et al. [2] proved that the stacking sequence contributes to delayed composite failure in natural and glass-fibre hybrid composites because specific stacking sequences improved the interlaminar shear strength, fracture toughness, and tensile strength. In the recent past, Feng et al. demonstrated that the mechanical properties of the hybridisation between pineapple leaf fibre (PALF) and Kevlar fibre depended on the fibre stacking sequence [3].

Fatigue failure is a significant problem encountered in structural components. Repeated dynamic loading below the ultimate tensile strength leads to internal damage accumulation over time, as shown by sudden and catastrophic failure. The study of fatigue failure behaviours is significant for various essential engineering applications. The fatigue failure behaviour is highly dependent on the stress distribution on the structural component, which principally depends on the laminate layering technique (stacking sequence and ply orientation) [4]. The variation in the laminate layering technique directly changes the fatigue stiffness and residual strength of the structural component [5,6]. The laminate layering techniques also affect the cyclic dissipation of energy during fatigue failure progression [7]. The cyclic dissipated energy is measured from the stress–strain hysteresis loop exhibited by the structural component [8,9]. Very few studies on the fatigue behaviour of hybrid composites (synthetic and natural fibres) with different stacking sequences have been reported in the literature. Previous studies by Sharba et al. [10] and Sivakumar et al. [11] analysed the fatigue life response of a glass-kenaf hybrid composite with different stacking sequences. Salman et al. discussed the fatigue failure of Kevlar–kenaf hybrid composites in different applications [12].

Relatively few studies have focused on the effect of the stacking sequence of hybridised PALF/carbon-fibre-reinforced composites on their fatigue behaviour. Different researchers and scientists have investigated the effect of ply orientation on fatigue behaviour. Most studies focused on a typical ply orientation, angle ply (0°/90°) and cross ply (± 45°), on the fatigue failure behaviour. Marine et al. [13] investigated the fatigue behaviour of angle-ply and cross-ply composites. In this study, the angle-ply composite exhibited a linear fatigue life trend, while the cross-ply composite exhibited a non-linear fatigue life trend. Quasi-symmetric ply orientation, which employs both ply orientations, is a recent and exciting research direction [14].

Very few investigations focusing on the fatigue behaviour response to the stacking sequence of hybrid laminate composites (synthetic and natural fibres), especially in quasi-symmetric ply orientations, are available in the literature. Moreover, the fatigue behaviour of PALF/carbon-fibre-reinforced epoxy remains unexplored. Therefore, this study explored the effects of the stacking sequence on the fatigue behaviour of PALF/carbon fibre hybrid composites.

## 2. Materials and Methods

### 2.1. Raw Materials

Table 1 shows the properties of the epoxy, PALF fibre (plain weave), and carbon fibre (plain weave) used in the laminates, which were locally sourced. The matrix material consisted of an epoxy polymer (ether of bisphenol A) and a hardener (triethylene tetra amine); both were mixed with an epoxy to hardener weight ratio of 100 : 27.8, as suggested by the manufacturer. All of the raw materials were supplied by Mecha Solve Engineering Sdn. Bhd., Selangor, Malaysia. Analysis of the chemical composition of PALF is shown in Table 2. Before the PALF was used, it was treated by soaking in a 5% alkali (NaOH) solution for 3 h with a 40:1 liquor ratio [15]. The alkali solution removed the surface impurities and hemicelluloses of PALF [16]. The treated PALF was dried for 8 h at 60 °C and further dried at room temperature for another 24 h.

### 2.2. Fabrication Method

PALF/carbon hybrid laminate composites were fabricated with the vacuum infusion technique. PALF and carbon plies were laminated in a glass mould by infusing epoxy resin into the lamination plies with a high vacuum pump (AST 22, AIRSPEC, Kuala Lumpur, Malaysia). The specimens were then cured in the mould for 12 h at room temperature. Finally, the laminate underwent a 2 h post-curing process in an oven at 80 °C with air circulation [21]. Table 3 shows the six different PALF and carbon layering sequences of the laminates prepared for this investigation. Each laminate consisted of 8 plies. The notation consists of two parts and is separated by “_”; the first part represents material stacking sequence, and the second part represents ply orientation.

### 2.3. Evaluation Methods

#### 2.3.1. Void Content

Void formation in the laminates during fabrication, due to entrapped gases, is difficult to avoid. Voids affect the mechanical properties of the laminates and increase the tendency for the physical properties to degrade due to moisture absorption in the laminates. Thus, void content determination is required to verify whether the fabrication process is suitable. The densities of the laminates (five samples of each laminate) were measured using a high-accuracy electronic densimeter (Qualitest MDS-300, Lauderdale, FL, USA; 0.0001 g/cm^3^ accuracy), FL, US. Equations (1) and (2), corresponding to ASTM D2734-09, were used to calculate the void content of the laminates.

(1) $$V\%=\frac{\rho_{t}-\rho_{e}}{\rho_{t}}\times 100.\;$$

(2) $$\rho_{t}=\frac{100}{\left(w_{f1}/\rho_{f1})+(w_{f2}/\rho_{f2})+(w_{p}/\rho_{p}\right)}$$

where ρ_f1_ is the PALF density, ρ_f2_ is the carbon fibre density, ρ_p_ is the epoxy polymer density, ρ_t_ is the laminate density, w_f1_ is the weight percentages of the PALF, w_f2_ is the weight percentages of the carbon fibre, w_p_ is the weight percentages of the epoxy polymer, and V% is the void content.

#### 2.3.2. Tensile Strength

The tensile properties of the laminates were evaluated using a universal testing machine (Instron-3369, Norwood, MA, USA) in compliance with ASTM D3039-76. For this purpose, 150 mm × 25 mm laminate samples were prepared. Their thickness depended on the layering sequence. The test gauge length was fixed to 100 mm. As suggested in the standard, the crosshead speed was set to a constant of 2 mm/min. Five samples from each stacking sequence were tested and the average values were reported.

#### 2.3.3. Fatigue Test

Fatigue tests were performed according to ASTM D3479-96 using a dynamic testing machine (Instron-8800 Servohydraulic, Norwood, MA, USA). The tests were conducted at 30, 45, 60, 75, and 90% of the ultimate tensile strength (UTS) at a frequency of 10 Hz [22,23,24,25]. Five samples from each stacking sequence were tested at each of the five stress levels. The fatigue tests ended when a failure occurred or when 10^6^ cycles were achieved. The results of the different specimens were then compared and analysed to evaluate their fatigue responses.

#### 2.3.4. Scanning Electron Microscopy (SEM)

The fracture surface morphology of the laminates was analysed using SEM (Hitachi TM3000, Tokyo, Japan). The fracture sections were coated with gold to capture better quality images. Scanning images were obtained at 100× and 500× magnifications using a 3–5 kV accelerating voltage.

## 3. Results

### 3.1. Void Content and Tensile Properties

The calculated void content of each PALF/carbon hybrid laminate composite, presented in Table 4, was below the allowable 5% limit, indicating an acceptable fabrication process [26]. The laminates with hybrid fibres showed slightly higher void contents than those of the neat PALF. The inhomogeneous fibre architecture caused air entrapment in the laminates owing to the non-uniform permeability of the fibre preform, which caused local variations in the resin velocity [27]. This local velocity variation was exacerbated by the microscale capillary effect.

**Figure 1:**
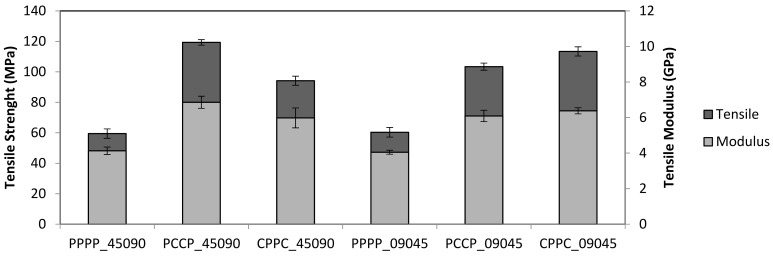
Tensile properties of the laminates.

Figure 1 presents the tensile strength and modulus of the laminates subjected to the quasi-static tensile tests. The tensile strength and modulus of PPPP_45090 and PPPP_09045 were almost similar, suggesting that a change in the stacking sequence contributed less to the tensile properties as the laminates were composed of the same material and the ply orientation was inversed to one another [28]. A single material laminate provides a stable and sound load transfer between the ply layers owing to the uniform mechanical properties of the material. However, the tensile strength of the laminate with an internal carbon ply was significantly different from the stacking orientation change, with PCCP_45090 achieving a higher tensile strength (119.34 MPa) than PCCP_09045 (103.43 MPa). The tensile modulus of PCCP_45090 (6.86 GPa) and PCCP_09045 (5.98 GPa) exhibited the same trend. This finding is consistent with previous studies in which the overall tensile performance was highly dependent on the strength of the core or the internal fibre in the laminate structure [29]. A study by Sezgin and Berkalp [30] also found that the adhesion interface bonding between the matrix is promising when carbon fibre was present in the core or interior.

In contrast, of the laminates with external carbon, CPPC_09045 yielded a higher tensile strength (113.43 MPa) than CPPC_45090 (94.18 MPa), while the tensile moduli of CPPC_09045 and CPPC_45090 were 6.38 GPa and 5.98 GPa, respectively. From these observations, it can be deduced that when the fibres with higher mechanical properties are aligned in the direction of the tensile load, a higher tensile strength will be achieved. In this case, carbon fibre has superior mechanical properties to PALF, and the fibres were oriented at 90°, aligning the fibres in the direction of the force and therefore causing the tensile strength to be higher than the other stacking sequences [31].

### 3.2. Wöhler Curves

This study aimed to understand the combined effect of the stacking sequence and ply orientation on the fatigue response of quasi-laminates. The change in the fatigue life of the laminates was identified and discussed by comparing the average number of cycles as a function of the UTS ratio, as listed in Table 5. As expected, the fatigue results demonstrate that for each type of composite and for a given high fatigue stress value (0.6 UTS and above), the fatigue life of the hybrid laminates (PCCP_45090, CPPC_45090, PCCP_09045, and CPPC_09045) was considerably higher than that of the non-hybrid laminates (PPPP_45090 and PPPP_09045). This indicates that the hybridisation process positively affected the fatigue strength. Moreover, for a given number of fatigue failure cycles, PCCP_45090 and CPPC_09045 exhibited a considerably higher useful life than the other laminates. However, the PCCP_45090 laminate exhibited a considerably better lifespan for every UTS ratio than CPPC_09045, especially at a low UTS ratio.

**Figure 2:**
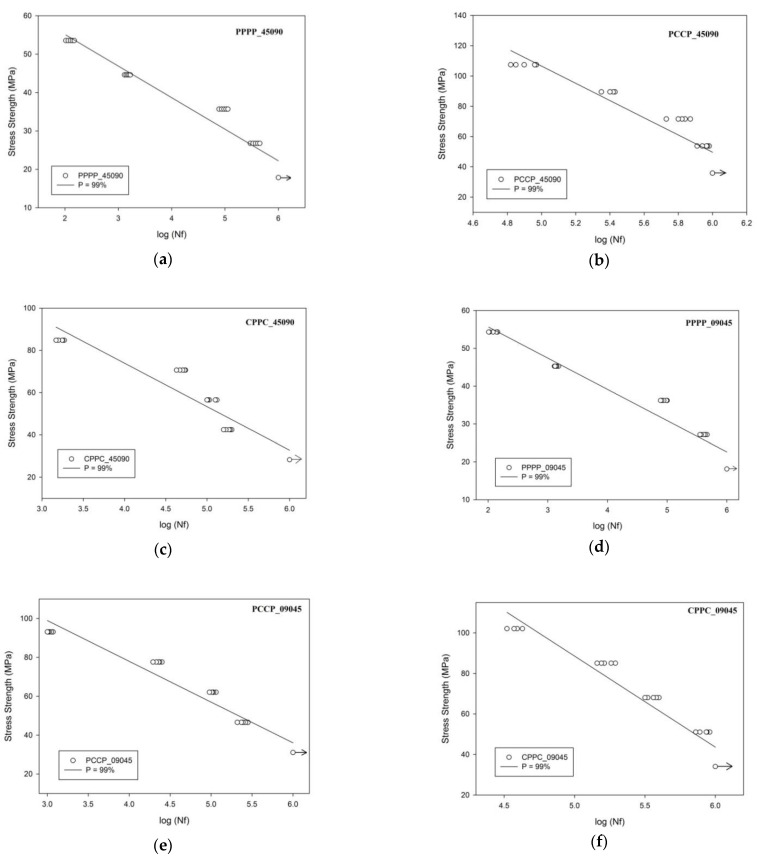
Experimental results and P–S–N for the laminates; (**a**) PPPP_45090, (**b**) PCCP_45090, (**c**) CPPC_45090, (**d**) PPPP_09045, (**e**) PCCP_09045 and (**f**) CPPC_09045.

To compare the fatigue sensitivity of the stacking sequences and ply orientation of the laminates, the stress strength against the number of cycles was plotted with a regression line at the 99% confidence level, as shown in Figure 2. The endurance limit, fixed at one million cycles, and the unbroken samples are indicated with an arrow notation. Based on the plotted regression, the line-modified Wöhler’s law (Equation (3)) was used to model the life of the specimens [25]:

(3) $$\log\left(N_{f}\right)=A-B\cdot \sigma_{x}+C\cdot s$$

where A and B represent the intrinsic parameters of the tested material, $\sigma_{x}$ is the axial maximum loading stress, and C is the number of standard deviations (s) corresponding to the specified confidence level. C equals -2, corresponding to Wöhler’s curve with a 99% survival probability [25]. Table 6 summarises the identified values for the parameters of Equation (3), and Figure 2 shows the corresponding curves. The linear regression coefficient (R) approached 1, which indicated the suitability of the model to the experimental data.

**Figure 3:**
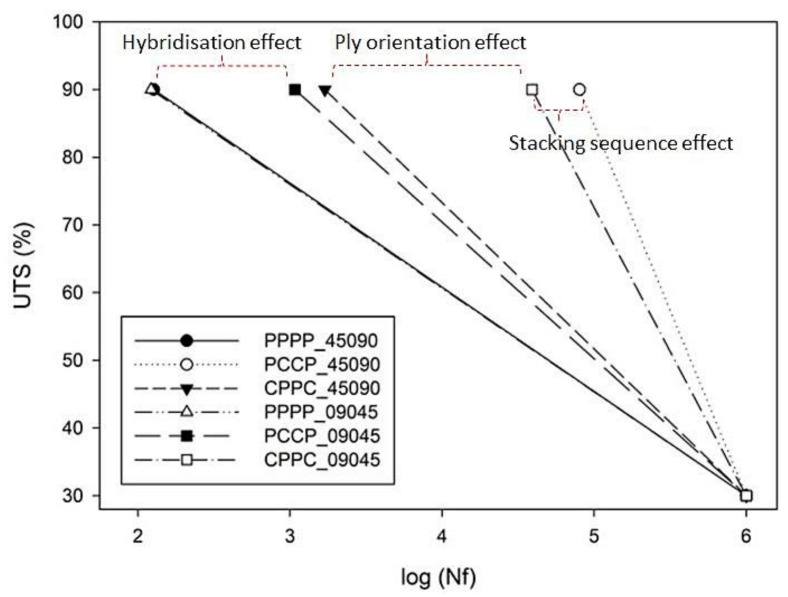
Plots normalised to UTS as a function of the life cycles.

Figure 3 shows the normalised Wöhler curves plotted using Equation (3) with the data recorded in Figure 2. The *x*-axis reports the decimal logarithm of the number of cycles to failure for each sample, while the *y*-axis represents the percentage of the maximum applied stress and the tensile strength. The data represent the non-failure samples, shown by an arrow notation. The positive effect of hybridisation was observed when comparing the normalised S–N curves for the laminates in the figure. The hybrid laminates exhibited a higher fatigue strength and resistance than the non-hybrid laminates. Several researchers have suggested that hybridisation contributes to variations in mechanical and physical properties [32]. It is clear that hybridisation allows for a higher number of cycles to failure than the single fibre laminates, especially at higher applied stress. This is mainly due to the superior tensile strength of the carbon fibres [33]. However, the endurance performance decreased after the hybridisation process. This phenomenon represents the slower fatigue stiffness degradation of the non-hybrid laminate, as compared to that of the hybrid laminate. This phenomenon is due to the excellent ability of plant fibres to absorb and deflect cracks through their complex microstructure, as compared to synthetic fibres. The same behaviour trend has been identified in other hybrid composites in the literature [10].

By comparing PCCP_45090 with CPPC_09045 in Figure 3, it is clear that the different stacking sequences significantly affected the fatigue strength. Both were composed of carbon fibre ply oriented longitudinally to the loading direction. Several researchers have demonstrated that assigning a superior material as an internal layer or core improves the fatigue strength and life of the laminate [34]. Micro-cracking occurs from the outer layer to the middle layer because the pre-stress is exerted on the outer layer of the specimen [25]. Sezgin and Berkalp [30] reported a strong adhesion between the carbon ply and the matrix, and strong bonding at the interface imparts good fatigue strength to the laminates.

However, PCCP_09045 and CPPC_09045 exhibited a different trend. PCCP_09045 exhibited a lower fatigue life than CPPC_09045, that is, the laminate with the internal carbon ply had a lower fatigue life than the external carbon ply laminate. This finding contradicts the earlier results because the fatigue life for the carbon ply in the ± 45° orientation was considerably lower than that in the 0°/90° orientation [35]. The ± 45° ply undergoes off-axis effects, such as off-axis matrix cracking, which can degrade the laminate stiffness [36]. This finding is in good agreement with the classical laminate theory, which has been discussed in previous studies [37]. In contrast, previous studies confirmed that the carbon ply orientation manipulated the overall mechanical properties of hybrid laminates [38]. It can be concluded that the changes in the fatigue strength due to the changes in the stacking sequence are less significant than those introduced by modifying the ply orientation. Consequently, the fatigue strength and fatigue endurance increased by changing the ply orientation towards the force loading direction.

### 3.3. Hysteresis Energy

**Figure 4:**
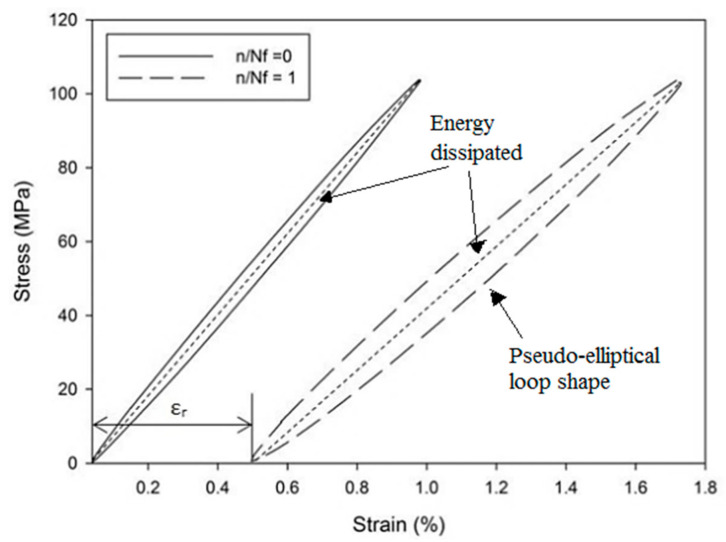
First and final hysteresis loop of PCCP_09045 tested at 0.75 UTS.

**Figure 5:**
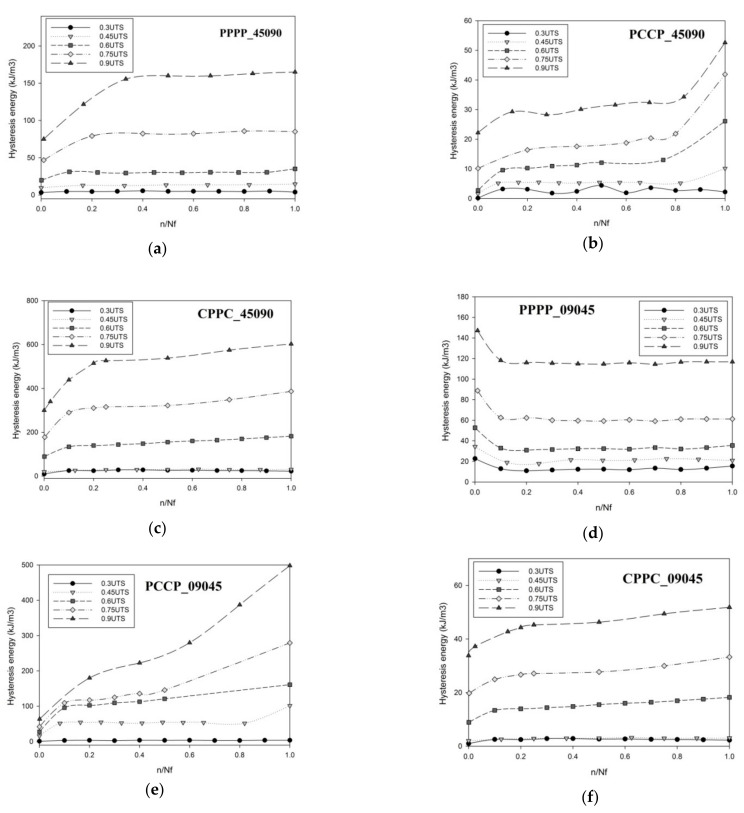
Hysteresis energy for the laminates produced in this study; (**a**) PPPP_45090, (**b**) PCCP_45090, (**c**) CPPC_45090, (**d**)PPPP_09045, (**e**) PCCP_09045 and (**f**). CPPC_09045.

Figure 4 shows the typical stress–strain hysteresis loops of the first (n/N_f_ = 0) and last loading cycles (n/N_f_ = 1) of PCCP_09045 at a 0.75 UTS ratio. These loops evolved from lower to higher strains at a constant stress level with creep-like behaviour. This is due to the fatigue loading consisting of a non-zero static load superimposed with sinusoidal variations for a zero loading ratio. The hysteresis loops drifted away from each other along the displacement axis with a pseudo-elliptical loop shape. The trend followed an exponential behaviour, with the lower exponents corresponding to the highest number of cycles, N. The area enclosed by the individual hysteresis loops presented inelastic damage or energy dissipation during a given cycle. Figure 5 shows the evolution of the hysteresis energy as a function of the life ratio for the PALF/carbon hybrid laminate composites, in which the hysteresis energy corresponds to the area enclosed within the loops, as shown in Figure 4. The enclosed area was numerically calculated using a simple trapezoidal summation of the area.

In general, the dissipated energy increased as the load was applied, as shown in Figure 5. Almost all laminates exhibited zero energy dissipation at a 0.3 UTS strength ratio because no breakage occurred during the test. The highest energy dissipation ranges were recorded by CPPC_45090 and PCCP_09045, in which the highest level was 602.7 and 498.1 kJ/m^3^ at a 0.9 UTS strength ratio, respectively. Concurrently, the lowest energy dissipation ranges were recorded by PCCP_04509 and CPPC_09045, in which the highest level was 52.6 and 51.83 kJ/m^3^ at a 0.9 UTS strength ratio, respectively. The results demonstrate that the laminates with the carbon ply oriented at ±45° developed a considerably higher energy dissipation than the 0°/90° laminates. Previous studies have demonstrated that laminates with the carbon ply oriented at ±45° have a lower fracture toughness than laminates with carbon ply oriented at 0°/90° [25,28]. The laminates with the ply oriented at ±45° failed because of pure in-plane shear due to the sliding and realignment of the fibre ply in the load direction. The same energy dissipation trend was observed for flax–epoxy composites [25].

Figure 5 shows that PPPP_45090 and PPPP_09045 exhibited two distinct phases of energy dissipation evolution. The first phase rapidly increased during the initial cycles of PPPP_45090, while PPPP_09045 exhibited the opposite. The second phase exhibited a stabilised region, where no significant energy dissipation dependence was observed against an increasing number of cycles for both laminates. This situation explains the significant effect of the different ply orientations on energy dissipation. Previous studies indicated that angle-ply laminates showed an incremental energy dissipation as the life cycle increased, while cross-ply laminates showed an inverse trend [39]. The finding was blended with the typical fatigue damage (matrix cracking), usually initiated from the exterior surface before propagating through the entire laminate [40]. In the second phase, the energy dissipation showed a stable trend at a certain level until the laminates fractured. During this phase, the load slowly transferred to the entire laminate structure before failure [10]. The transition between the phases became expeditious as the stress strength ratio increased for PPPP_09045, whereas PPPP_45090 showed the opposite trend.

Figure 5 shows that the internal carbon ply laminates, PCCP_45090 and PCCP_09045, exhibited three distinct energy dissipation evolution phases. During the initial cycles of the first phase, the energy dissipation increased. During the second phase, a gradual steady-state increasing trend was visible. The energy dissipated during the third phase increased sharply up to the point of failure of both specimens owing to the interaction between the two different material properties in the laminates after the hybridisation process. Similar findings were reported by Ribeiro et al., who found that the interaction between two different material properties affects energy dissipation during fatigue testing [41]. PCCP_09045 exhibited a substantially higher energy dissipation level than PCCP_45090 because of the lower fracture toughness for the inner carbon ply oriented at ± 45° [25,28]. However, the external carbon ply laminates, CPPC_45090 and CPPC_09045, exhibited only two district phases. The phenomenon can be explained by the carbon ply being assigned as the external layer. The superior strength of the carbon fibre [42] eliminated the PALF strength because fatigue failure was initiated at the external layer [40], and consequently, only two identical energy dissipation phases were exhibited.

### 3.4. Stiffness Evolution

**Figure 6:**
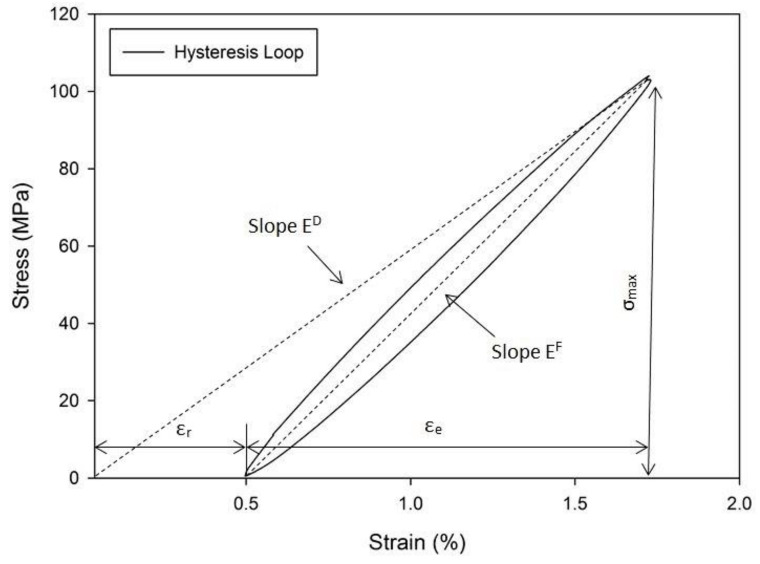
Final hysteresis loop of PCCP_09045 tested at 0.75 UTS.

**Figure 7:**
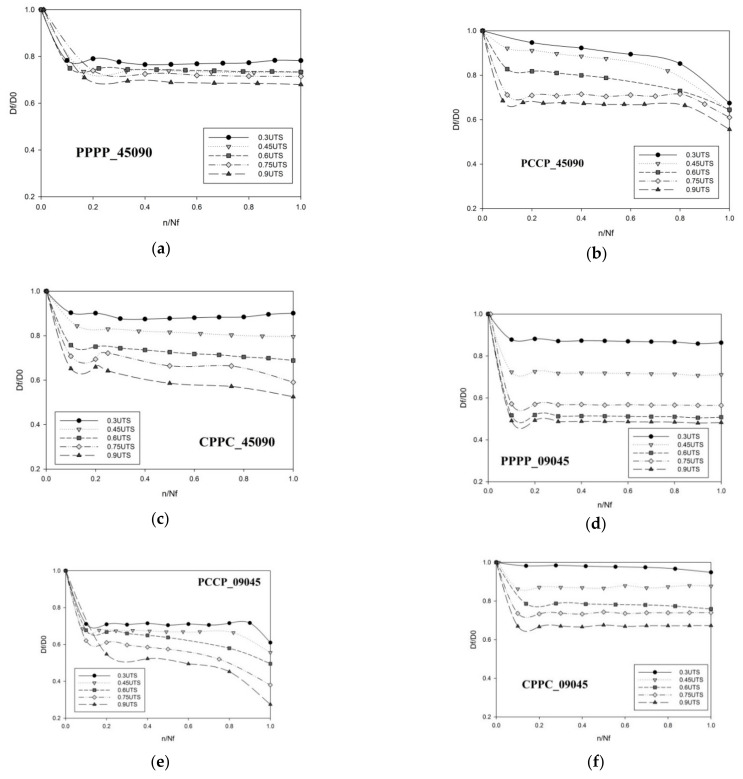
Dynamic modulus of the laminates produced in this study; (**a**) PPPP_45090, (**b**) PCCP_45090, (**c**) CPPC_45090, (**d**) PPPP_09045, (**e**) PCCP_09045 and (**f**). CPPC_09045.

Figure 6 presents the typical stress–strain hysteresis loop of the last loading cycles (n/N_f_ = 1) of PCCP_09045 at a 0.75 UTS ratio. The cyclic dynamic modulus (E^D^) corresponds to the slope of the hysteresis loop axis. Generally, the slope becomes less steep with an increasing number of cycles, which degrades the modulus caused by internal damage development [43]. Figure 7 presents the curves of the evolution of the normalised dynamic modulus, which is the ratio of the cyclic dynamic modulus (E^D^) to the measured modulus during the first cyclic dynamic modulus ($E_{0}^{D}$).

Figure 7 shows that PPPP_45090, CPPC_45090, PPPP_09045, and CPPC_09045 exhibited a 10% to 60% stiffness decrease in the early loading cycles and continued to gradually decrease until failure occurred. Conversely, the stiffness of PCCP_45090 and PCCP_09045 decreased in three stages—namely, an initial rapid stiffness reduction, followed by an intermediate step with a steady but slow decrease, and a final rapid decline before sample failure. This response was due to the superior properties of the internal carbon ply, which was manifested by the sudden total degradation of the dynamic modulus [34]. Previous researchers reported a similar three-stage dynamic modulus degradation with respect to the behaviour of materials loaded in fatigue [44]. The variation in the dynamic modulus degradation stages indicates that the material stiffening phenomenon is influenced by the material stacking sequence [25].

In Figure 7, the dynamic modulus of PPPP_09045 decreased to 50%, as compared to PPPP_45090 (20–25%). Based on previous studies, the loading stress was concentrated at the internal layer (core) of the laminates during the fatigue test [34]. The internal layer of PPPP_09045 was oriented at ±45°, which was off-axis to the load direction. Consequently, the microfibrills inside the fibres began straightening and tended to reorient themselves after each cycle to naturally align with the load direction. This phenomenon caused the dynamic modulus of the laminates to degrade as the load strength increased.

### 3.5. Damage Mechanism

**Figure 8:**
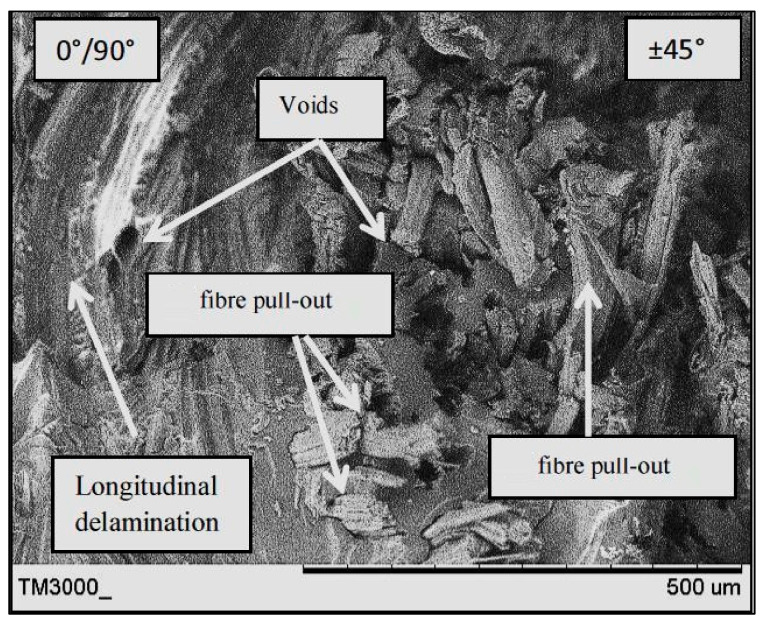
SEM image of PPPP_09045 at a 45% UTS ratio.

**Figure 9:**
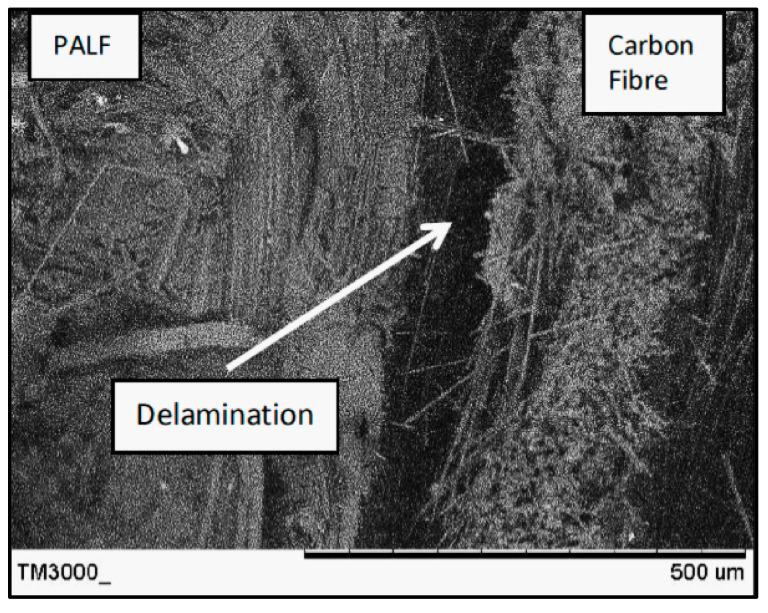
SEM image of CPPC_45090 at a 45% UTS ratio.

**Figure 10:**
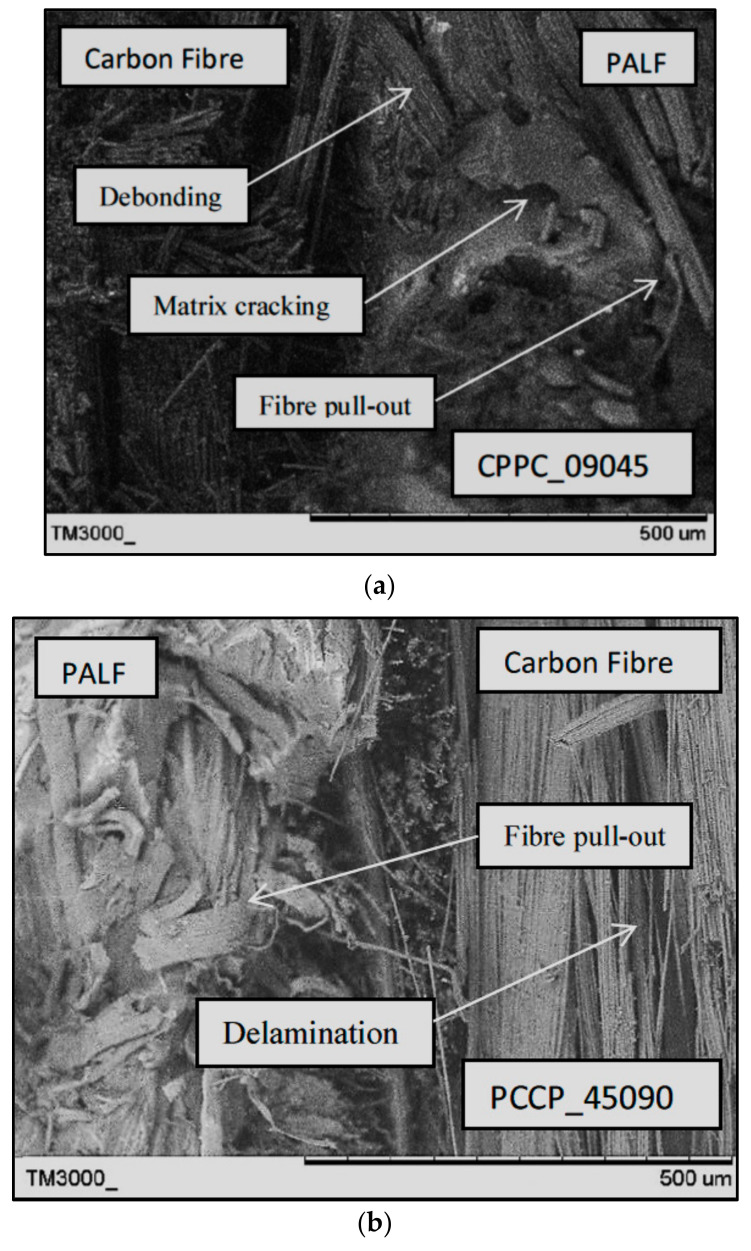
SEM images of; (**a**) CPPC_09045 and (**b**) PCCP_45090 at a 45% UTS ratio.

Figure 8, Figure 9 and Figure 10 present the fracture surface morphologies of the PALF/carbon hybrid laminate composites after fatigue failure. Figure 8 presents an SEM image of PPPP_09045 at a 45% UTS ratio, demonstrating PALF fibre pull-out and debonding with the epoxy matrix. Fractures, created by the frictional shear stress, developed after delamination was completed through the debonded interface during the debonding process [28]. The development of frictional shear stress by the pull-out mechanism contributed to the energy dissipation capacity [45]. The laminate exhibited low toughness properties because of the formation of voids after fibre pull-out. The same finding was reported by Seghini et al., who compared non-hybrid basalt–epoxy composites and hybrid flax–basalt–epoxy composites [35]. The non-hybrid composite mainly failed due to fibre pull-out fracture after the fatigue test. Moreover, the image shows longitudinal fibre–matrix delamination along the laminate width, which is a failure caused by delamination of the fibre at a 0° orientation after undergoing perpendicular axial loading. Large quantities of fibre tearing were also observed in the longitudinal fibre matrix, indicating good interfacial bonding between the fibre and matrix.

Figure 9 presents an SEM image of CPPC_45090 at a 45% UTS ratio after fatigue failure. The laminate exhibited a poor interface between the carbon fibre and PALF, causing delamination between the plies. Delamination can usually happen due to the loss of adhesion between two adjacent plies. In certain situations, delamination fracture occurs at the interface between layers with different fibre orientations and fibre materials, as shown in Figure 9. The delamination effect worsens when there are significant differences in the mechanical properties of the two corresponding layers, leading to the creation of out-of-plane stresses.

Figure 10 presents SEM images of CPPC_09045 and PCCP_45090 at a 45% UTS ratio. The two different stacking sequences and ply orientation techniques created mixed fracture behaviour for both laminates. The CPPC_09045 image shows that the carbon fibre was blunted at the PALF/matrix interface during the early cracking stages. The damage was propagated to the interior PALF layers until they failed primarily from debonding and fibre pull-out. The PCCP_45090 PALF layer failed by the long fibre pull-out mode at first, before propagating into the internal carbon layer. The internal carbon layer exhibited delamination failure at the region between the carbon/PALF interface and the carbon/carbon interface, demonstrating the better ductility of the carbon layers. PCCP_45090 underwent the typical composite fatigue failure stages where the failure was initiated and propagated from the external to the internal layers [46]. A similar longitudinal fibre–matrix delamination failure mode was identified for the 0°/90° layer in both CPPC_09045 and PCCP_45090.

## 4. Conclusions

The effects of the stacking sequence on the fatigue behaviour of PALF/carbon hybrid laminate composites were investigated. The laminates were produced with different stacking sequence configurations using the vacuum infusion technique. The laminates were evaluated through a series of mechanical tests, fatigue tests, and morphological observations. The following conclusions can be drawn from this study:

1. PCCP_45090 recorded the maximum tensile strength and modulus. This is because the carbon ply direction was aligned with the loading direction. The carbon ply is an internal layer which promises good adhesion between the matrix. The superior properties of the carbon fibre dominated the overall tensile properties of the laminate.
2. The fatigue response investigation indicated that PCCP_45090 and CPPC_09045 yielded higher useful lives than the other laminates, especially when the laminates are subjected to high-intensity stress levels. The normalised stress against the number of cycles showed that the stacking sequence of different ply orientations significantly affected the fatigue behaviour, as compared to the stacking sequences of the material. The stacking sequences of the laminates significantly affected the hysteresis energy evolution. The hysteresis energy tended to follow the ply orientation of the carbon fibre, with higher hysteresis energy being exhibited for the laminates with the carbon ply off-axis to the load direction. The measured stiffness evaluation exhibited a considerable effect when the carbon ply was oriented at ± 45°.
3. Morphological analyses indicated that the laminates which failed under fatigue testing failed in the typical modes of fibre pull-out, fibre breakage, delamination, debonding, and matrix cracking. Failure during fatigue was initiated from the external layer, as shown by fibre pull-out, matrix cracking, and fibre breakage. When the external layer was a carbon ply, delamination was the primary failure mode.

## Figures and Tables

**Table 1:** Mechanical properties of the constituent fibres and matrix polymers.

Property	Epoxy	Fibre	
		PALF	Carbon Fibre
Tensile strength (MPa)	45	148.44	3530
Tensile modulus (GPa)	1.75	10.46	230
Strain at failure (%)	6	1.05	1.5
Reference	[17]	[18]	[19]

**Table 2:** Chemical composition of PALF [20].

Composition	Percentage (%)
Cellulose	47.74
Hemicellulose	15.98
Lignin	2.44

**Table 3:** Ply orientation and stacking sequence of the laminates: PALF (P) and carbon fibre (C).

Orientation	Layering Sequence (Number of Layers)		Notation
	External	Internal	
[± 45°_2_, 0°/90°_2_]_s_	PALF (8)		PPPP_45090
	PALF (4)	Carbon (4)	PCCP_45090
	Carbon (4)	PALF (4)	CPPC_45090
[0°/90°_2_, ± 45°_2_]_s_	PALF (8)		PPPP_09045
	PALF (4)	Carbon (4)	PCCP_09045
	Carbon (4)	PALF (4)	CPPC_09045

**Table 4:** Void contents of the laminates. The standard deviations are presented in brackets.

Laminate	Void Content (%)
PPPP_45090	2.63 (± 0.85)
PCCP_45090	2.02 (± 0.55)
CPPC_45090	2.23 (± 0.53)
PPPP_09045	2.53 (± 0.76)
PCCP_09045	2.03 (± 0.41)
CPPC_09045	1.53 (± 0.32)

**Table 5:** Average number of cycles as a function of the UTS ratio. The standard deviations are presented in brackets.

Laminates	0.9 UTS	0.75 UTS	0.6 UTS	0.45 UTS	0.3 UTS
PPPP_45090	127 (± 16)	1495 (± 122)	95,391 (± 9160)	372,791 (± 44,672)	> 1 × 10^6^
PCCP_45090	80,109 (± 8157)	253,994 (± 18,290)	652,772 (± 79,452)	898,846 (± 82,191)	> 1 × 10^6^
CPPC_45090	1693 (± 60)	49,758 (± 3967)	113,350 (± 14,008)	180,579 (± 16,328)	> 1 × 10^6^
PPPP_09045	122 (± 15)	1380 (± 97)	88,924 (± 8,130)	408,251 (± 44,507)	> 1 × 10^6^
PCCP_09045	1076 (± 62)	22,458 (± 1,943)	104,638 (± 7,392)	209,154 (± 42,153)	> 1 × 10^6^
CPPC_09045	38,948 (± 3,675)	167,822 (± 18,672)	357,677 (± 30,992)	829,277 (± 69,979)	> 1 × 10^6^

**Table 6:** Fitted data for the Wöhler’s law parameters and the linear regression coefficient.

	A	B	s	R
PPPP_45090	7.8344	0.1216	0.433	0.9696
PCCP_45090	6.3915	0.0364	0.869	0.9175
CPPC_45090	4.5662	0.0486	1.5128	0.9431
PPPP_09045	7.8736	0.1209	0.4301	0.9703
PCCP_09045	5.5684	0.0477	1.0747	0.9711
CPPC_09045	6.9463	0.0436	1.3128	0.9640

## Data Availability

The data presented in this study are available on request from the corresponding author.
